# Neutrophilic eccrine hidradenitis following dasatinib initiation in a patient with chronic myeloid leukemia

**DOI:** 10.1016/j.jdcr.2025.03.033

**Published:** 2025-05-08

**Authors:** Avery Watson, Noah Roberts, Caitlin Noble, Hannah Badon, Allison Cruse

**Affiliations:** a School of Medicine, The University of Mississippi Medical Center, Jackson, Mississippi; b Dermatologist and Dermatopathologist, Greenville, South Carolina; c Department of Dermatology, The University of Mississippi Medical Center, Jackson, Mississippi; d Department of Dermatology, Department of Pathology, The University of Mississippi Medical Center, Associate Professor, Jackson, Mississippi

**Keywords:** drug reaction, neutrophilic eccrine hidradenitis, neutrophils, oncodermatology

## Introduction

Neutrophilic eccrine hidradenitis (NEH) is a benign entity which typically occurs in patients with leukemia undergoing chemotherapy. While the most common culprit regimen is cytarabine followed by daunorubicin, this eruption may also be associated with numerous other chemotherapies.^1^ Clinically, NEH presents heterogeneously as erythematous, edematous papules or plaques that are painful or painless, often with a predication for the trunk.^1^ The differential diagnosis is most often cellulitis or infection, but definitive diagnosis is made through biopsy.^2^^,^^3^ Histologically, the entity is characterized by necrosis of eccrine glands and ducts and often features a neutrophilic infiltrate, although in severely neutropenic patients, this infiltrate may not be exuberant.^2^

## Case report

**Fig 1:**
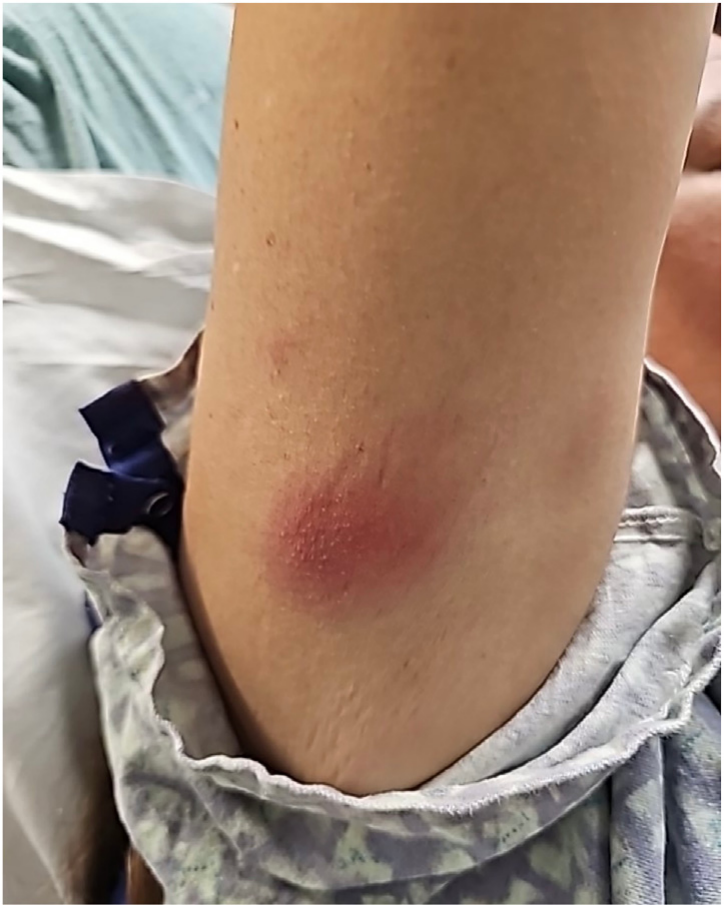
Poorly circumscribed erythematous nodules with palpable induration on R posterior upper arm.

A 48-year-old female with a history of chronic myeloid leukemia (CML), inflammatory arthritis, and chronic obstructive pulmonary disease was admitted to a tertiary care center with complaints of abdominal pain, bone pain, mouth sores, and fever approximately 7 days after transitioning from imatinib 400 mg daily to dasatinib 100 mg daily due to treatment nonresponse. The patient disclosed the development of a painful, warm rash located on the right posterior arm (Fig 1) and right breast that appeared 3 days after dasatinib initiation. Notably, she did not experience cutaneous complications during imatinib use. She was ultimately diagnosed with febrile neutropenia and treated with broad spectrum antibiotics: vancomycin, cefepime, and metronidazole. Dasatinib was held. Imaging of abdomen, pelvis, and gallbladder were within normal limits. Additionally, soft tissue ultrasound of the right upper arm revealed diffuse edema of the subcutaneous tissues without focal fluid collection. All blood and urine cultures, as well as antinuclear antibody were negative.

**Fig 2:**
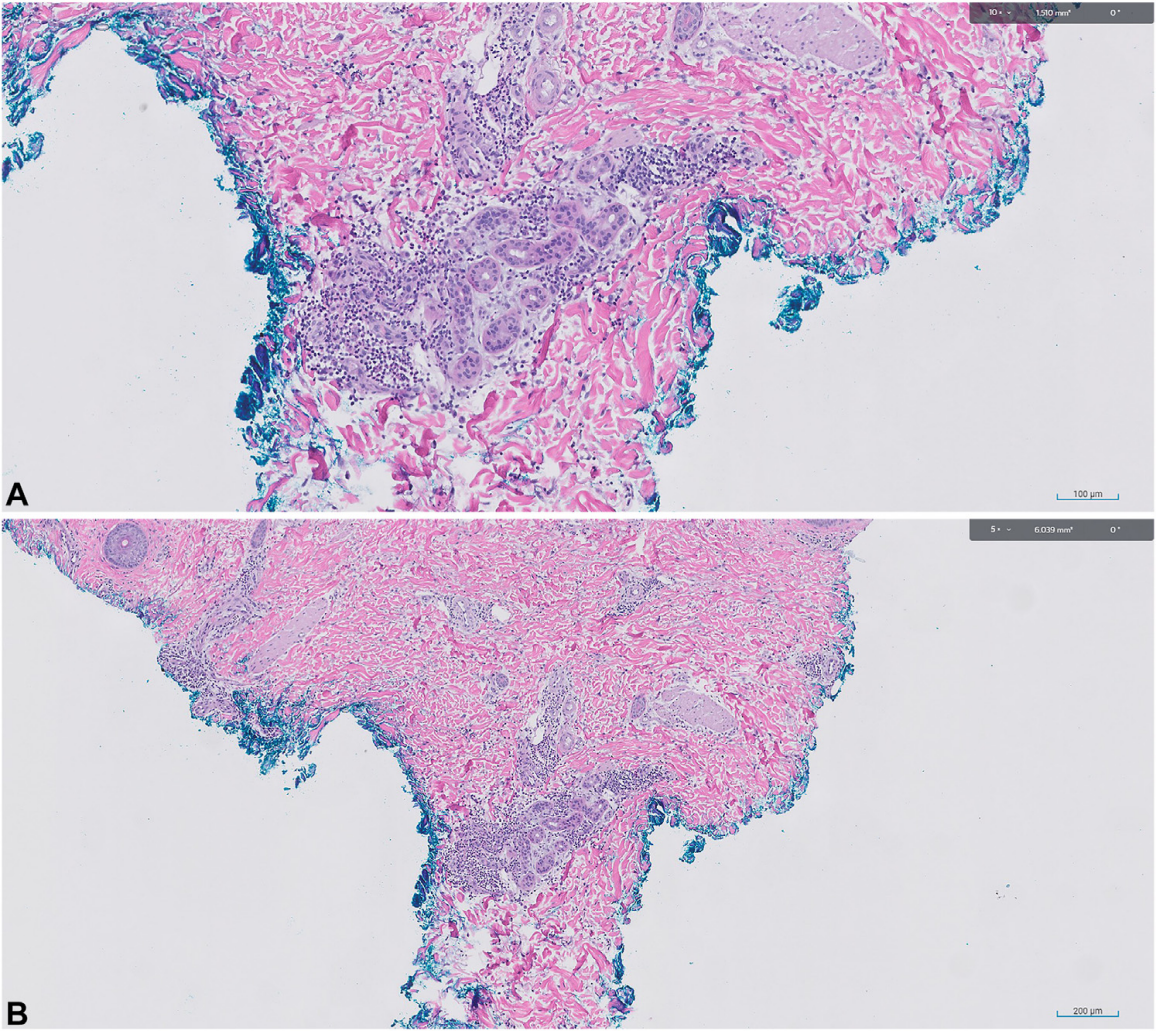
**A,** and **B,** Histology from a punch biopsy of the R posterior arm reveals a slightly acanthotic epidermis with mild spongiosis. A superficial and somewhat deep perivascular infiltrate is noted. A neutrophilic infiltrate is centered around the eccrine glands, which demonstrate mild squamous metaplasia.

The dermatology service was consulted for further workup due to concern for skin infection. On examination, edematous, erythematous nodules with induration were noted to the right posterior upper arm, right posterior shoulder, right breast, and right superior back. 2 punch biopsies were obtained from the right posterior upper arm for histopathology and tissue culture. Tissue culture revealed growth of staphylococcus epidermidis, thought to be a contaminant or colonizing organism. Histopathology (Fig 2 revealed an acanthotic epidermis with mild spongiosis along with a superficial and deep lymphocytic, perivascular infiltrate. A neutrophilic infiltrate was noted surrounding eccrine glands which demonstrated mild squamous metaplasia. Gram stain was negative for bacterial cocci, and periodic acid Schiff plus diastase and Grocott methenamine silver stains were negative for yeast forms and fungal hyphae. Acid fast bacilli stain was negative for acid-fast bacilli.

The findings were most suggestive of NEH. Symptomatic treatment with triamcinolone 0.1% cream twice daily was initiated and the appearance of the rash and associated pain continued to improve. At discharge, the patient’s antibiotic coverage deescalated to doxycycline 100 mg twice daily for 7 days and dasatinib was discontinued. Cessation of dasatinib resulted in complete resolution of skin findings, as noted at subsequent hematology visits.

## Discussion

NEH is typically associated with hematologic malignancies such as acute myeloid leukemia and CML, but may also be associated with various infections and inflammatory conditions ranging from lupus to Behcet disease.^3^ It is believed that our patient most likely developed NEH induced by dasatinib, a member of the broader class of chemotherapy agents known as tyrosine kinase inhibitors (TKIs) that act as small-molecule ATP-competitive inhibitors of SRC and ABL tyrosine kinases.^4^

The mechanism surrounding the development of NEH is currently not well understood; however, current literature suggests 3 general processes underlie NEH eruptions: altered expression of inflammatory effector molecules, abnormal neutrophil function, and genetic predisposition. Specifically, TKIs may interfere with neutrophilic signal transduction cascades, or drug excretion through sweat may result in eccrine gland cell death, inducing neutrophil chemotaxis.^3^, ^4^, ^5^, ^6^Alternatively, NEH may be a hypersensitivity reaction residing on the spectrum of other neutrophilic dermatoses often associated with hematological malignancies, including Sweet syndrome and pyoderma gangrenosum.^3^^,^^5^

Because NEH typically manifests heterogeneously with a fever and rash, differential is broad and infection is often suspected.^1^ Eruptions begin an average of 10 days after chemotherapy initiation, with 70% of cases occurring after the first course of chemotherapy.^1^^,^^3^ The time course of eruptions confounds diagnosis, as vasculitis, Stevens Johnson Syndrome, and phototoxic eruptions are also likely diagnoses in this patient population.^6^ Notably, out patient’s first eruption occurred 4 months from initiation chemotherapy but only 3 days after new agent initiation, highlighting the importance of time to eruption in clinical suspicion for NEH. Although the typical presentation of NEH does involve a sterile neutrophilic infiltrate on histology, the presence of gram-positive cocci on culture, as in our case, does not exclude NEH as a diagnosis.^2^ The potential for NEH to be culture-positive affirms the necessity of biopsies in differentiating between NEH and infection. A skin biopsy demonstrating a dense neutrophilic infiltrate of the eccrine unit with necrosis and dermal edema is requisite for diagnosis of NEH.^6^

The lesions of NEH often resolve within weeks following discontinuation of the causative medication, but corticosteroids, antibiotics, and nonsteroidal anti-inflammatory drugs have been found to be helpful in symptomatic management.^5^ TKIs like dasatinib should be discontinued when grade 3 adverse events (limiting activities of daily living, prolongation of hospitalization) are experienced, while grade 1 events (no intervention) allow for a repeated trial of TKIs at a reduced dose.^4^^,^^7^ Current literature acknowledges 35% of patients receiving dasatinib will experience a cutaneous reaction; however, such reactions are primarily mild or moderate (urticaria, photosensitivity, nail or pigment changes).^8^^,^^9^ Notably, dasatinib is a newer class of TKI that is considered exempt from desensitization protocols previously established due to concerns for Stevens-Johnson syndrome in imatinib-treated CML patients.^9^ If cessation of chemotherapy is not possible, dapsone has been successfully used in the prevention of future NEH and can be considered for pretreatment before rechallenging.^5^^,^^10^ Ultimately, NEH treatment is supportive and involves multidisciplinary teams to minimize cutaneous reactions while maximizing oncologic outcomes.

In conclusion, NEH is a benign necrosis of eccrine sweat glands and ducts typically associated with hematologic malignancies that has a heterogeneous clinical presentation. Given the clinical resemblance to infection, thorough evaluation and biopsy are requisite for proper diagnosis. To our knowledge, we report the first case of dasatinib-induced NEH, increasing awareness of causative agents of NEH to prevent unnecessary antibiotic use or alterations to chemotherapy regimens.

## Conflicts of interest

None disclosed.
